# Cigarette and IL-17A synergistically induce bronchial epithelial-mesenchymal transition via activating IL-17R/NF-κB signaling

**DOI:** 10.1186/s12890-020-1057-6

**Published:** 2020-01-30

**Authors:** Libing Ma, Ming Jiang, Xiaoli Zhao, Jingyi Sun, Qilu Pan, Shuyuan Chu

**Affiliations:** 1 Department of Respiratory and Critical Care Medicine, Affiliated Hospital of Guilin Medical University, Guilin, 541001 Guangxi China; 2 Laboratory of Respiratory Disease, Affiliated Hospital of Guilin Medical University, Guilin, 541001 Guangxi China

**Keywords:** Epithelial-mesenchymal transition, Bronchial epithelial cell, IL-17, NF-κB, Cigarette smoke extract

## Abstract

**Background:**

IL-17A directly induces epithelial-mesenchymal transition (EMT) in alveolar epithelial cells. It could coordinate with cigarette smoke extract (CSE) to promote proliferation of bronchial epithelial cells. In this study, we aim to explore the direct effect of IL-17A and CSE on EMT in bronchial epithelial cells.

**Methods:**

Bronchial epithelial cells were isolated from C57BL/6 mice, and cocultured with CSE or/and IL-17A. E-cadherin and Vimentin expressions in cells were detected using immunofluorescence staining. IL-17R expression was detected using immunohistochemistry staining. NF-κB expression was assessed using western blotting. When NF-κB was inhibited by BAY 11–7821, expressions of NF-κB, E-cadherin and Vimentin were measured.

**Results:**

The protein expression of E-cadherin in bronchial epithelial cells was lowest in CSE + IL-17A group, followed by CSE group. In contrast, the protein expression of Vimentin was highest in CSE + IL-17A group, followed by CSE group. Similarly, IL-17R and NF-κB expressions were highest in CSE + IL-17A group, followed by CSE group and IL-17A group. NF-κB inhibitor could inhibit the expressions of E-cadherin and Vimentin.

**Conclusions:**

Cigarette and IL-17A could synergistically induce EMT in bronchial epithelial cells through activating IL17R/NF-κB signaling. Our findings contribute to a better understanding in airway EMT and pathogenesis of respiratory diseases, which are involved IL-17A and cigarette smoking. Those will provide novel avenues in the immunotherapy of lung diseases.

## Background

Epithelial-mesenchymal transition (EMT) in bronchial epithelial cells is involved in pathogenesis of lung cancer, chronic obstructive pulmonary disease (COPD), asthma and pulmonary fibrosis, which all have a high prevalence [1–4]. IL-17A is involved in these lung diseases. This interleukin promotes chronic immune inflammation, contributing to occurrence and development of those lung diseases [5–7]. In airway, IL-17A could induce production of chemokine and cytokines from bronchial epithelial cells [8, 9]. Interestingly, IL-17A not only promotes airway remodeling [10], but also directly induces EMT in alveolar epithelial cells [11]. That indicates a direct effect of IL-17A on structure cells in airway. However, there is rare report about the direct effect of IL-17A on EMT in bronchial epithelial cells. Thus, we investigated the direct effect of IL-17A on EMT in murine bronchial epithelial cells.

As widely accepted, cigarette smoking is a risk factor for lung diseases, particularly lung cancer and COPD. Cigarette smoke extract (CSE) could coordinate with IL-17A to induce proliferation of human bronchial epithelial cell line 16HBE [12]. CSE could significantly enhance IL17R expression in 16HBE as well [12]. Those findings suggest the synergistical effect of CSE and IL-17 on bronchial epithelial cells. Since CSE could induce EMT of bronchial epithelial cells in vitro and in vivo [13], we explored the synergistical effect of CSE and IL-17 on EMT in murine bronchial epithelial cells. Furthermore, previous study found that NF-κB activation could promote EMT in human bronchial epithelial cells [14]. Thus, we investigated the role of NF-κB signaling in the effect of CSE and IL-17 on bronchial EMT as potential mechanism in this study.

## Methods

### Mouse cell preparation

Female C57BL/6 mice (7–8 weeks old) were purchased from Hunan SJA Laboratory Animal Co. Ltd. (Hunan, China). They were housed in cages with free access to water and standard mouse chow. Animal protocols were approved by the Institutional Animal Care and Use Committee of Guilin Medical University and conformed to National Institutes of Health guidelines for the use of rodents. All experiments were repeated three times.

Mice were anaesthetized using 2% isoflurane inhalation and killed by cervical dislocation. The mouse bronchus was separated from lobes of lung, and then was minced to small pieces and digested by 0.05% pronase (Sigma, MA, USA) in DMEM/F12 media (Invitrogen, CA, USA) at 4 °C overnight. Digestion was stopped using FBS (Gibco, CA, USA). The bronchial epithelial cells were identified by CK-18 immunofluorescence staining.

Primary bronchial epithelial cells were cultured for 24 h, and then cocultured with cigarette smoke extract (CSE) or/and IL-17A. CSE was prepared using a modification as previously reported [15]. In CSE group, bronchial epithelial cells were induced using 20% CSE. In IL-17A group, the cells were induced using 50 ng/ml IL-17A (Biolegend, CA, USA). In CSE + IL-17A group, the cells were cocultured with 20% CSE and 50 ng/ml IL-17A (Biolegend, CA, USA). Cells in all groups were cultured at 37 °C with 5%CO2 for 72 h. The cells without administration of CSE or IL-17A were control group.

### NF-κB signaling inhibition

NF-κB inhibitor, BAY 11–7821 (A4210, APEXBIO, Texas, USA), was used to inhibit NF-κB signaling. When primary bronchial epithelial cells were isolated and cultured for 24 h, they were cocultured with 10uM BAY 11–7821 overnight. Following that, the cells were prepared as described for IL-17A group, CSE group, CSE + IL-17A group and controls. The inhibition of NF-κB was identified using western blotting.

### Western blot analysis

The bronchial epithelial cells were treated with 200 μl RIPA for 10 min on ice, and then centrifuged at 12000×g (4 °C) for 15 min. The loaded proteins (150–170 μg) were separated on a 10% SDS-polyacrylamide gel electrophoresis, followed by transferring onto PVDF membranes. The samples were blocked with TBS-Tween 20 containing 5% skim milk for 60 min at room temperature, followed by at 4 °C overnight, and finally 30 min at room temperature. The membranes were incubated with rabbit anti-mouse antibodies against mouse NF-κB (0.5 μg/ml, ab16502; Abcam, Cambridge, UK), or mouse anti-mouse β-actin antibody (1:5000, 60,008–1-Ig; Proteintech, IL, USA) for 90 min at room temperature. When washed with phosphate buffered saline, they were incubated with horseradish peroxidase conjugated goat anti-rabbit (1:6000, SA00001–2; Proteintech, IL, USA) or anti-mouse antibodies (1:5000, SA00001–1; Proteintech, IL, USA) for 90 min at room temperature. At last, blots were developed with the ECL Plus reagents (Thermo pierce, IL, USA). The protein bands were analyzed using Quantity One software (Bio-Rad, CA, USA).

### Immunohistochemistry and immunofluorescence staining

For immunohistochemistry staining, the slides of cells were fixed by 4% paraformaldehyde for 30 min and incubated in 3% H2O2 for 10 min to quench endogenous peroxidase activity. The slides were incubated with primary rabbit anti-mouse antibody against IL-17R (1:50, ab180904; Abcam, Cambridge, UK) at 4 °C overnight, and then were incubated with horseradish peroxidase conjugated goat anti-rabbit IgG antibody (PV-9000, Zisbio, Beijing, China) at 37 °C for 30 min. After rinsing with PBS for three times, 3′3-diaminobenzidine-tetrahydrochloride was applied on the slides as a chromogen for 1–5 min. Slides were counterstained in haematoxylin for 5–10 min. Micrographs were obtained using a microscope (BA210T, motic, Xiamen, China).

For immunofluorescence staining, the slides of cells were fixed by 4% paraformaldehyde for 30 min, and incubated with 0.3% tritonX-100 at 37 °C for 30 min, then 5% bovine serum albumin. The slides were incubated with primary mouse anti-mouse Cytokeratin18 (1:50, ab668; Abcam, Cambridge, UK), mouse anti-mouse E-cadherin (1:50, ab76055; Abcam, Cambridge, UK), or rabbit anti-mouse antibody against Vimentin (1:50, ab92547; Abcam, Cambridge, UK) at 4 °C overnight, and then were incubated with Alexa Fluor 594-conjugated Goat Anti-Mouse IgG(H + L)(SA00006–3, Proteintech, IL, USA) or Alexa Fluor 594 -conjugated Goat Anti-Rabbit IgG(H + L)(SA00006–4, Proteintech, IL, USA). Finally, slides were staining with DAPI (4′,6-diamidino-2-phenylindole) at 37 °C for 10 min. Micrographs were taken from a microscope (BA210T, motic, Xiamen, China).

### Statistical analysis

Statistical analyses were performed using SPSS 21.0 (IBM SPSS Inc., Chicago, IL, USA). *P* values < 0.05 were considered to be statistically significant. Group data are expressed as the mean ± standard deviation (SD). Significant differences were evaluated using one-way analysis of variance (ANOVA) followed by the Student–Newman–Keuls test or the Games–Howell test.

## Results

### Cigarette and IL-17A synergistically induce IL-17R expression in bronchial epithelial cells

**Fig. 1:**
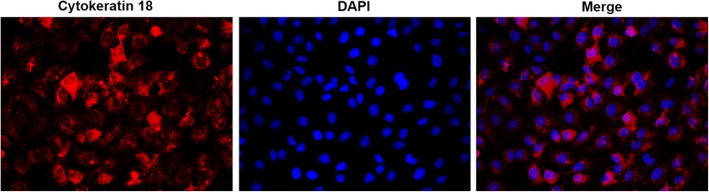
Cells identification. When bronchial epithelial cells were isolated and cultured, cells were identified by immunofluorescence staining of CK-18. Cells were mainly CK-18+ staining. (× 400 magnification)

Primary murine bronchial epithelial cells were identified using immunofluorescence staining of Cytokeratin18 (Fig. 1). Cytokeratin 18 is the bronchial epithelial autoantigen [16].

**Fig. 2:**
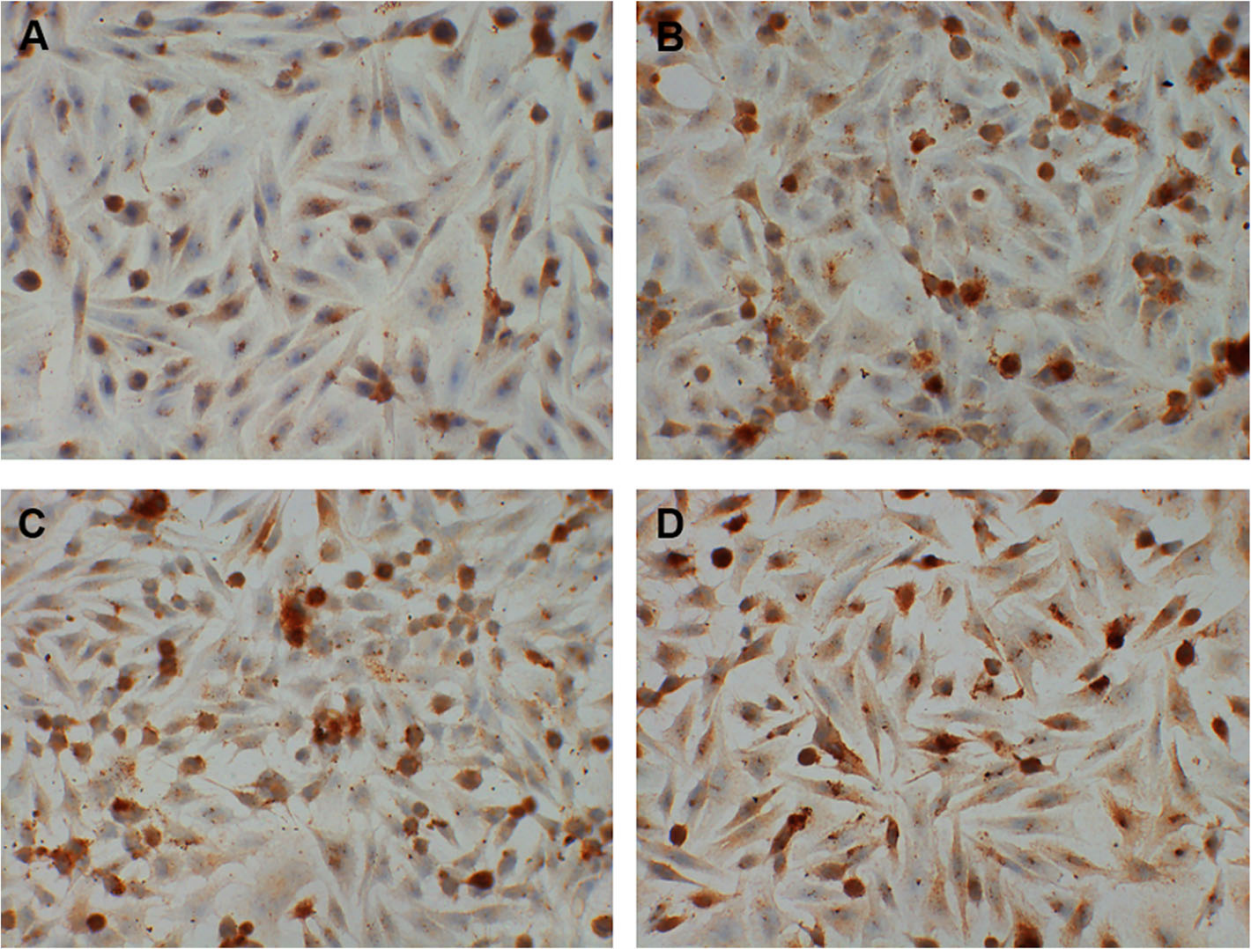
IL-17R expression in bronchial epithelial cells. When bronchial epithelial cells were stimulated by cigarette smoke extract (CSE) or/and IL-17A, IL-17R expression in cells were detected using immunohistochemistry staining. In CSE group and IL-17A group, IL-17R expression was increased when compared with controls. IL-17R expression was highest in CSE + IL-17A group. **a** control group. **b** CSE group. **c** IL-17A group. **d** CSE + IL-17A group. (× 400 magnification)

In murine bronchial epithelial cells, the expression of IL-17R was higher in CSE group and IL-17A group than controls. It’s highest in CSE + IL-17A group (Fig. 2). These results suggest that CSE or IL-17A could induce IL-17R expression in bronchial epithelial cells. Moreover, CSE could play a synergistical role with IL-17A in inducing the IL-17R expression.

### Cigarette and IL-17A synergistically stimulate activation of NF-κB

**Fig. 3:**
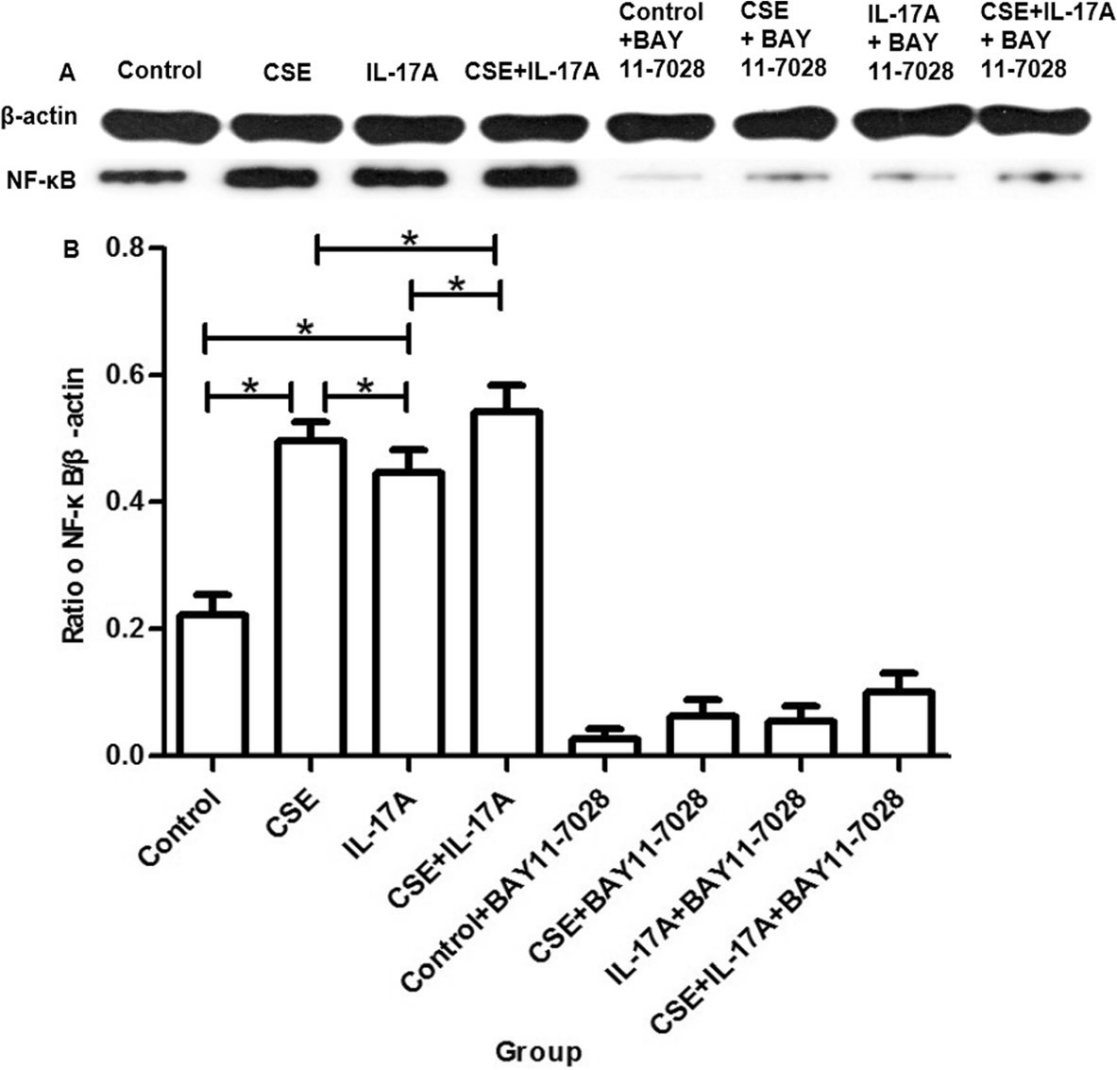
The protein expression of NF-κB in bronchial epithelial cells. Bronchial epithelial cells were inhibited NF-κB, and then stimulated by cigarette smoke extract (CSE) or/and IL-17A. NF-κB expression was measured using Western blotting. In CSE group and IL-17A group, NF-κB expression was increased when compared with controls. NF-κB expression was highest in CSE + IL-17A group. When NF-κB was inhibited, NF-κB expressions in all group were significantly reduced. **a** Western blotting. **b** Quantitation of protein bands

The protein expression of NF-κB in bronchial epithelial cells was higher in CSE group and IL-17A group than controls. It’s highest in CSE + IL-17A group (Fig. 3). These results suggest that NF-κB activation could be stimulated by CSE. And CSE could coordinate with IL-17A to stimulate NF-κB activation. When NF-κB in bronchial epithelial cells was inhibited by BAY 11–7821, NF-κB protein expression was significantly reduced (Fig. 3).

### Cigarette and IL-17A synergistically induce bronchial epithelial-mesenchymal transition through NF-κB signaling

**Fig. 4:**
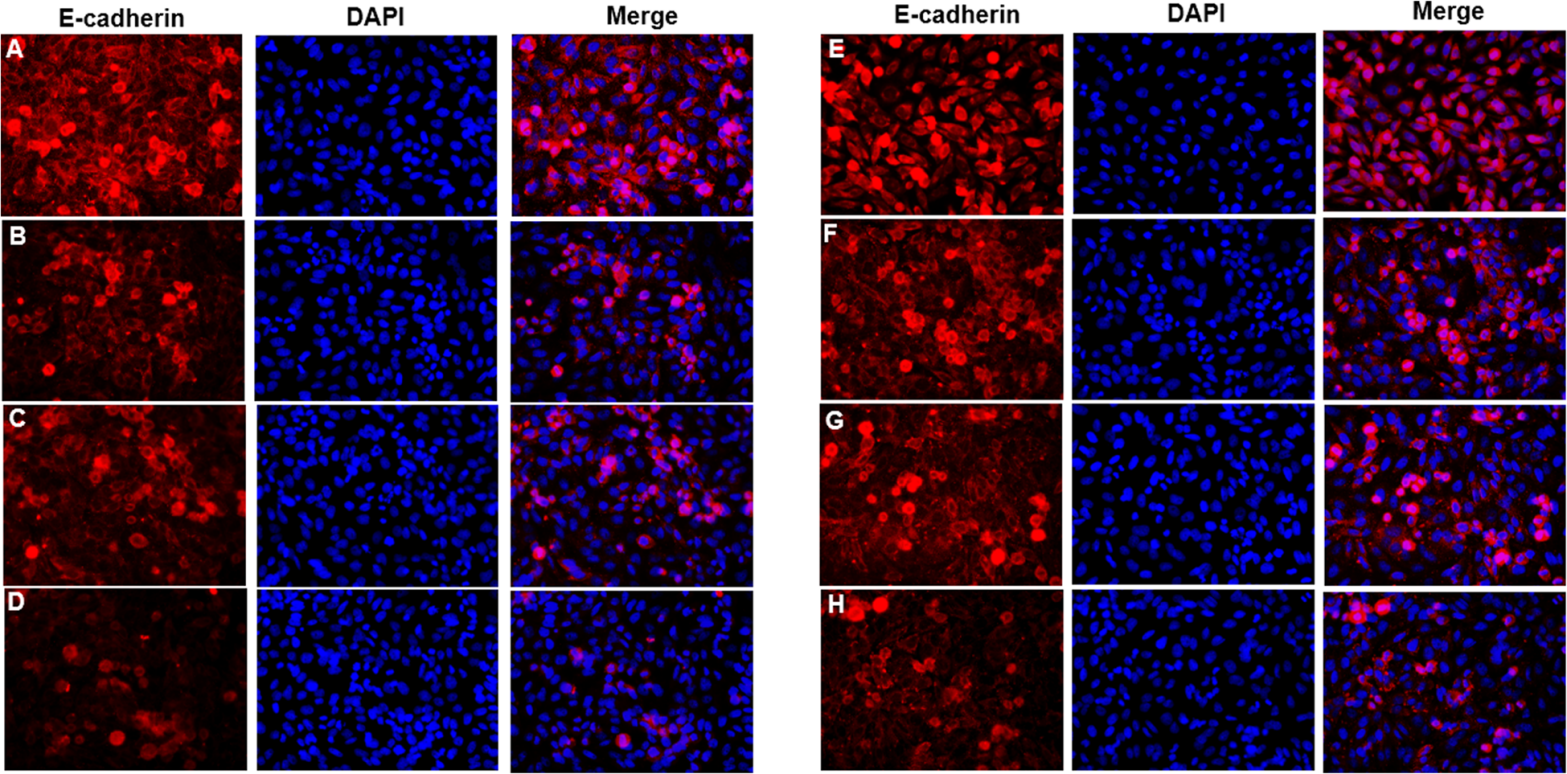
E-cadherin expression in bronchial epithelial cells. When bronchial epithelial cells were stimulated with cigarette smoke extract (CSE) or/and IL-17A, E-cadherin expression in cells was detected using immunofluorescence staining. E-cadherin expression in CSE group was lower than that in controls, and was lowest in CSE + IL-17A group. When NF-κB was inhibited, E-cadherin expression was increased in cells stimulated with CSE and CSE + IL-17A compared to those without inhibition. **a** E-cadherin expression in control group without NF-κB inhibition. **b** E-cadherin expression in CSE group without NF-κB inhibition. **c** E-cadherin expression in IL-17A group without NF-κB inhibition. **d** E-cadherin expression in CSE + IL-17A group without NF-κB inhibition. **e** E-cadherin expression in control group with NF-κB inhibition. **f** E-cadherin expression in CSE group with NF-κB inhibition. **g** E-cadherin expression in IL-17A group with NF-κB inhibition. **h** E-cadherin expression in CSE + IL-17A group with NF-κB inhibition. (× 400 magnification)

**Fig. 5:**
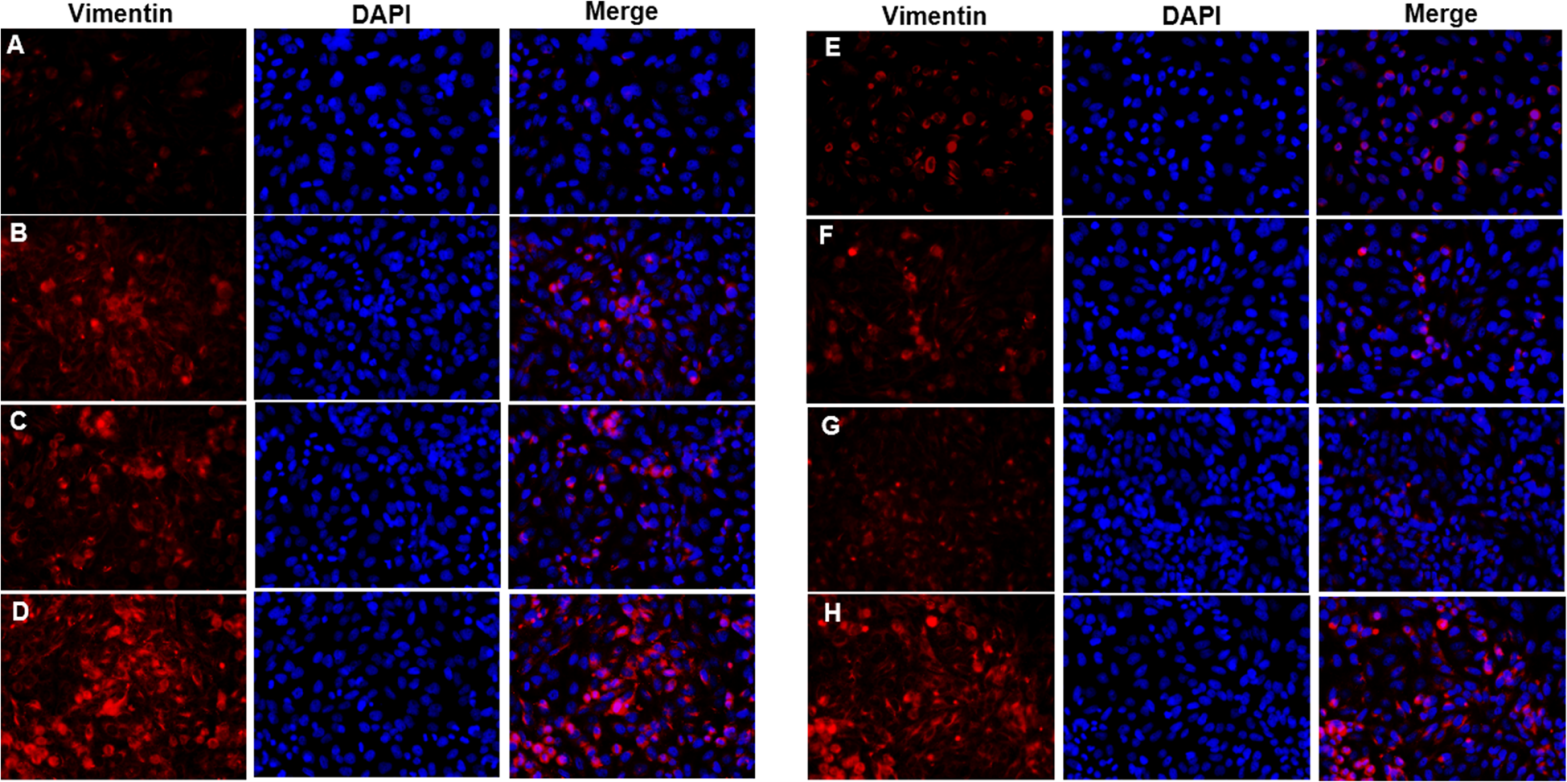
Vimentin expression in bronchial epithelial cells. When bronchial epithelial cells were stimulated with cigarette smoke extract (CSE) or/and IL-17A, Vimentin expression in cells was detected using immunofluorescence staining. Vimentin expression in CSE group was higher than that in controls, and was highest in CSE + IL-17A group. When NF-κB was inhibited, Vimentin expression was decreased in cells stimulated with CSE and CSE + IL-17A after NF-κB inhibited compared to those without inhibition. **a** Vimentin expression in control group without NF-κB inhibition. **b** Vimentin expression in CSE group without NF-κB inhibition. **c** Vimentin expression in IL-17A group without NF-κB inhibition. **d** Vimentin expression in CSE + IL-17A group without NF-κB inhibition. **e** Vimentin expression in control group with NF-κB inhibition. **f** Vimentin expression in CSE group with NF-κB inhibition. **g** Vimentin expression in IL-17A group with NF-κB inhibition. **h** Vimentin expression in CSE + IL-17A group with NF-κB inhibition. (× 400 magnification)

The expression of E-cadherin in bronchial epithelial cells was decreased in CSE group when compared with controls. E-cadherin expression was lowest in CSE + IL-17A group (Fig. 4a-d). In contrast, the expression of Vimentin in bronchial epithelial cells was increased in CSE group compared to controls, and was highest in CSE + IL-17A group (Fig. 5a-d). These results indicate that CSE could not only induce EMT in bronchial epithelial cells, but also act synergistically with IL-17A to promote that EMT.

When NF-κB was inhibited, the expressions of E-cadherin and Vimentin in bronchial epithelial cells were detected using immunofluorescence staining. With NF-κB inhibition, E-cadherin expression was increased in cells stimulated by CSE or CSE + IL-17A (Fig. 4e-h) compared to those without inhibition (Fig. 4a-d). In contrast, Vimentin expression was decreased in cells stimulated by CSE or CSE + IL-17A when inhibiting NF-κB (Fig. 5e-h), compared to those without NF-κB inhibition (Fig. 5a-d). These results indicate that when NF-κB is inhibited, the bronchial EMT is reduced. Thus, the CSE and IL-17A could synergistically induce EMT in bronchial epithelial cells through NF-κB signaling.

## Discussion

Our study demonstrates that the protein expression of E-cadherin in bronchial epithelial cells is lowest in CSE + IL-17A group, followed by CSE group. In contrast, the protein expression of Vimentin is highest in CSE + IL-17A group, followed by CSE group. Those could be inhibited by NF-κB inhibitor. In addition, the expressions of IL-17R and NF-κB are similar with that of Vimentin in bronchial epithelial cells. Our results suggest that cigarette and IL-17A could synergistically induce EMT through IL17R/NF-κB signaling in bronchial epithelial cells.

Our results showed that in CSE group, E-cadherin expression in bronchial epithelial cells was lower than controls, whereas Vimentin expression was higher than controls. Decreased E-cadherin expression and increased Vimentin expression could indicate EMT in bronchial epithelial cells [17]. Thus, our findings suggest that CSE could induce EMT in bronchial epithelial cells. Previous study reported that CSE could induce EMT of human bronchial epithelial cells and airway remodeling in animal model of COPD [13]. The results from our study are consistent with previous findings, and further confirm the effect of CSE on EMT in murine bronchial epithelial cells. Moreover, we found that in CSE + IL-17A group, E-cadherin expression was lower and Vimentin expression was higher than those in CSE group. Those findings indicate that cigarette and IL-17A could synergistically induce EMT in bronchial epithelial cells.

Furthermore, we explored the possible mechanism on the synergistical effect of CSE and IL-17A on EMT in bronchial epithelial cells. IL-17A could bind to IL-17R, and induce the activation of NF-κB and IL-17R expression in human bronchial epithelial cells, resulting in an increased production of cytokines, such as IL-19, CXCL-2, − 3 and − 5 [18]. Similarly, CSE could stimulate the activation of NF-κB in human bronchial epithelial cells, leading to inflammation, oxidative stress and even cell malignant transformation [19, 20]. Interestingly, in alveolar epithelial cells, CSE could coordinate with IL-17A to activate NF-κB, leading to inflammation [21]. Thus, we explored the role of IL-17R/NF-κB signaling in the synergistical effect of CSE and IL-17A on EMT in bronchial epithelial cells. Our study shows that IL-17R and NF-κB expressions in CSE group or IL-17A group were increased when compared with controls. In CSE + IL-17A group, IL-17R and NF-κB expressions were highest. Those findings confirm that CSE and IL-17A could synergistically activate IL-17R/NF-κB signaling in bronchial epithelial cells.

Moreover, we inhibited NF-κB to further demonstrate the role of NF-κB signaling in the effect of CSE and IL-17A on EMT in bronchial epithelial cells. Our study shows that in CSE group and CSE + IL-17 group, E-cadherin expression in bronchial epithelial cells was lower than controls, whereas Vimentin expression was higher. In CSE + IL-17E group, E-cadherin expression was lowest but Vimentin expression was highest. Those results suggest that EMT should be most aggravated when stimulated by CSE and IL-17A, followed by CSE stimulation alone. However, the EMT is not significantly different from controls when stimulated with IL-17A alone. Thus, CSE or CSE coordinated with IL-17A may induce EMT of bronchial epithelial cells via NF-κB activation. However, our study couldn’t conclude that IL-17A could induce EMT in murine bronchial epithelial cells via NF-κB activation, although IL-17A could stimulate IL-17R/NF-κB signaling in those cells. Interestingly, an increase of IL-17A promotes COPD, asthma and lung cancer, which all have a high prrevalence [6, 7, 22]. And cigarette smoking is widely accepted as a significantly risk factor for those diseases. Thus, our study may contribute to a better understanding in the pathogenesis of those respiratory diseases, which are involved both of IL-17A and cigarette smoking.

## Conclusions

Our study shows an increase of EMT in bronchial epithelial cells when stimulated with CSE, which was mostly aggravated when CSE combined with IL-17A. Similar expression pattern of IL-17R and NF-κB was observed. When NF-κB is inhibited, EMT stimulated by CSE alone or combined with IL-17A was significantly reduced. Those suggest that cigarette and IL-17A could synergistically induce EMT in bronchial epithelial cells through activating IL17R/NF-κB signaling. Our findings contribute to a better understanding in airway EMT and the pathogenesis of respiratory diseases, which are involved with IL-17A and cigarette smoking. Those will provide novel avenues in the immunotherapy of lung diseases.

## Data Availability

The datasets used and/or analyzed during the current study are available from the corresponding author on reasonable request.

## Abbreviations

ANOVA: Analysis of variance
COPD: Chronic obstructive pulmonary disease
CSE: Cigarette smoke extract
EMT: Epithelial-mesenchymal transition
SD: Standard deviation
